# In vitro competition between two transmissible cancers and potential implications for their host, the Tasmanian devil

**DOI:** 10.1111/eva.13670

**Published:** 2024-03-10

**Authors:** Anne‐Lise Gérard, Rachel S. Owen, Antoine M. Dujon, Benjamin Roche, Rodrigo Hamede, Frédéric Thomas, Beata Ujvari, Hannah V. Siddle

**Affiliations:** ^1^ School of Life and Environmental Sciences Deakin University Waurn Ponds Victoria Australia; ^2^ CREEC/MIVEGEC, CNRS, IRD Université de Montpellier Montpellier France; ^3^ School of Biological Sciences University of Southampton Southampton UK; ^4^ Institute for Life Sciences University of Southampton Southampton UK; ^5^ The Roslin Institute The University of Edinburgh Edinburgh UK; ^6^ School of Natural Sciences University of Tasmania Hobart Tasmania Australia

**Keywords:** competition model, Lotka–Volterra model, Tasmanian devil, transmissible cancer

## Abstract

Since the emergence of a transmissible cancer, devil facial tumour disease (DFT1), in the 1980s, wild Tasmanian devil populations have been in decline. In 2016, a second, independently evolved transmissible cancer (DFT2) was discovered raising concerns for survival of the host species. Here, we applied experimental and modelling frameworks to examine competition dynamics between the two transmissible cancers in vitro. Using representative cell lines for DFT1 and DFT2, we have found that in monoculture, DFT2 grows twice as fast as DFT1 but reaches lower maximum cell densities. Using co‐cultures, we demonstrate that DFT2 outcompetes DFT1: the number of DFT1 cells decreasing over time, never reaching exponential growth. This phenomenon could not be replicated when cells were grown separated by a semi‐permeable membrane, consistent with exertion of mechanical stress on DFT1 cells by DFT2. A logistic model and a Lotka–Volterra competition model were used to interrogate monoculture and co‐culture growth curves, respectively, suggesting DFT2 is a better competitor than DFT1, but also showing that competition outcomes might depend on the initial number of cells, at least in the laboratory. We provide theories how the in vitro results could be translated to observations in the wild and propose that these results may indicate that although DFT2 is currently in a smaller geographic area than DFT1, it could have the potential to outcompete DFT1. Furthermore, we provide a framework for improving the parameterization of epidemiological models applied to these cancer lineages, which will inform future disease management.

## 1 INTRODUCTION

Cancer is a diverse group of diseases resulting from the loss or gain of function in proteins regulating cell division, growth and death which can lead to the formation of a tumour (Gatenby & Brown, 2020; Hanahan & Weinberg, 2011; Vincze et al., 2022). While cancer is the second leading cause of human deaths worldwide (Sung et al., 2021), this disease is not restricted to humans; cancer or cancer‐like phenomena have been found in multicellular organisms across the tree of life (Madsen et al., 2017; Vincze et al., 2022). Before eventually causing host death, cancer often influences ecological interactions by altering an individual's competitive ability, vulnerability to predators, and/or susceptibility to pathogens (Vittecoq et al., 2013). As such, cancer can limit population growth and cause population declines (McAloose & Newton, 2009), yet research into the impact of cancers on wild populations remains relatively underdeveloped (Hamede et al., 2020). Indeed, monitoring the occurrence and prevalence of cancer in wildlife is challenging as affected hosts are often preyed upon or die undetected (Vittecoq et al., 2013).

Tasmanian devils (*Sarcophilus harrisii*) have undergone an extensive population decline owing to a transmissible cancer, devil facial tumour disease (first described as DFTD and also named DFT1; Pearse & Swift, 2006). First reported in 1996 in north‐eastern Tasmania (Hawkins et al., 2006), the disease likely emerged in the 1980's (Stammnitz et al., 2023) and has since spread to most of the island, decimating devil populations (Cunningham et al., 2021). DFT1 tumours develop predominantly around the head and oral cavity (Pye, Woods, & Kreiss, 2016). These tumours progressively impair feeding, metastasize and hijack host resources, ultimately causing death within 2 years from infection (Wells et al., 2017, 2019). Unlike most cancers, DFT1 cells are directly transmitted between individuals as allografts (Pearse & Swift, 2006), spreading from host to host akin to a parasite (Ujvari, Gatenby, & Thomas, 2016). Transmission occurs through biting, which is frequent during the mating season, when feeding and during other social interactions (Hamede et al., 2013).

**FIGURE 1:**
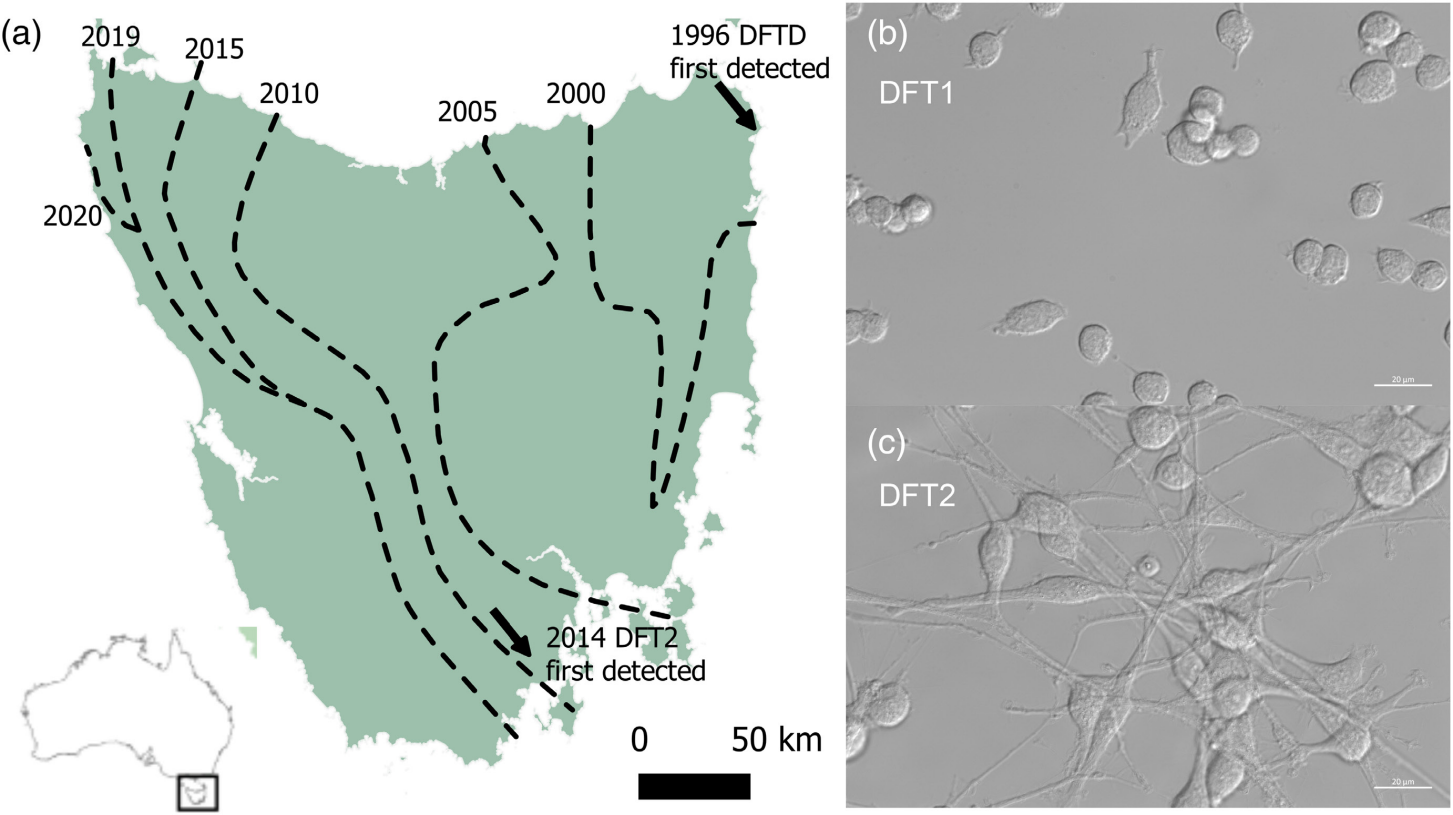
(a) Distribution of DFT1 and DFT2 in Tasmania (Australia) from 1996 until 2020. DFT1 was first reported in north‐eastern Tasmania in 1996 and DFT2 in the south‐eastern d'Entrecasteaux Peninsula in 2014. Dashed lines represent the spread of DFT1, while DFT2 is still confined to south‐eastern Tasmania (reproduced with permission from (Belkhir et al., 2022)). (b) DFT1 4906 and (c) DFT2 RV cell line morphologies, phase contrast imaging (630×). DFT1 cells are rounder than DFT2 cells which show a neuronal‐like morphology.

Over the last 25 years, the overall number of devils has been reduced by 68% (Cunningham et al., 2021). Initial epidemiological modelling suggested that DFT1 might drive Tasmanian devils to extinction (McCallum et al., 2009), but more recent individual‐based models predict that devils will coexist with DFT1 (Wells et al., 2019), and several tumour regressions have been documented in wild animals (Margres et al., 2018; Pye, Hamede, et al., 2016). This is likely due to the emergence of host phenotypic and genetic adaptations in response to DFT1 (Epstein et al., 2016; Jones et al., 2008; Stahlke et al., 2020; Ujvari, Hamede, et al., 2016). However, the emergence of DFT2, a second fatal transmissible cancer symptomatically similar but genetically distinct to DFT1, could challenge the species' survival (Pye, Hamede, et al., 2016). Although transmissible cancers have also been reported in dogs (Murgia et al., 2006) and bivalves (Metzger et al., 2016), the Tasmanian devil is the only mammalian species known to be affected by two independent transmissible tumours. DFT2 is estimated to have emerged in south‐eastern Tasmania in 2011, inside the d'Entrecasteaux Peninsula (Figure 1a; Stammnitz et al., 2023). While DFT1 progressed through Tasmania's landscape at a rate of 25 km/year, occupying now >95% of its host geographic range, DFT2 is spreading north of the Peninsula at a rate of 7 km/year (James et al., 2019). DFT1 and DFT2 co‐occur within the d'Entrecasteaux Peninsula, and three cases of individual co‐infection with DFT1 and DFT2 have been documented (R. Hamede, personal communication; James et al., 2019; Kwon et al., 2018). These co‐infection cases are characterized by distinct DFT1 and DFT2 tumours located in proximity of one another on the face, and/or in the oral cavity of the animals (James et al., 2019; Kwon et al., 2018). A majority of DFT tumours manifest on the face of devils, and hence, it is not unusual for multiple tumours of same and/or different origins and types (DFT1 and/or DFT2) to develop in close proximity (James et al., 2019). Habitat fragmentation and competition between both cancers are thought to be responsible for DFT2's slower movement across the landscape (James et al., 2019). Hence, the competitive potential of both tumours is key to predict whether DFT2 has the potential to propagate through the already weakened devil population.

In ecological systems, competition between species sharing a habitat is often modelled using the Lotka–Volterra equations for interspecific competition. Lotka–Volterra models can predict if species are likely to outcompete one another or if they will coexist (Hanahan & Weinberg, 2011; Lotka, 1910; Novoa‐Muñoz et al., 2021; Volterra, 1927). Competition outcome is influenced by the species' growth dynamics which can be summarized by two parameters: population growth when conditions are ideal (growth rate, density‐independent), and equilibrium population density when nutrients and space become limited (carrying capacity, density‐dependent; Freischel et al., 2021). However, competition outcome not only depends on a species' phenotype but also on the phenotype of its competitors, such as a competitor's ability to take up a certain resource. This is referred to as frequency‐dependent effects which can be measured using competition assays in which two cell types are grown together using different starting ratios (Freischel et al., 2021). While the Lotka–Volterra equations have historically been used to study competition in model organisms and natural systems (Gause & Witt, 1935; Moth & Barker, 1977; Park, 1954), they have also recently been applied to study competition between cancer cells (Farrokhian et al., 2022; Freischel et al., 2021). Although the use of ecological models is becoming more frequent in human cancer research (Amend & Pienta, 2015; Belkhir et al., 2021; Boutry et al., 2022; Korolev et al., 2014), no study has so far attempted to apply them to transmissible cancers. The DFT1‐DFT2 system presents an ideal and novel platform to apply this approach to understand the fundamental biological drivers of these fascinating diseases.

Here, we assess the competitive abilities of DFT1 and DFT2 using an in vitro co‐culture system adapted from (Freischel et al., 2021) combined with a modelling framework to predict competition outcomes. We also assess the fitness of each cell line in monoculture as no previous study has established the intrinsic growth dynamics of these tumours in vitro. Using this unique study system, we show that competition and more complex interactions arise when cancer becomes transmissible and exploits the same host species in the wild. Modelling the trajectory of these diseases throughout the Tasmanian devil population will be essential to better understand epidemiological dynamics and develop appropriate disease management and conservation strategies.

## 2 MATERIALS AND METHODS

### 2.1 Cell cultures

Representative cell lines for DFT1 (4906, also known as 88 (Pyecroft et al., 2007)) and DFT2 (RV also known as TD467 or 202T1 (Pye, Pemberton, et al., 2016) were cultured as previously described (Pye, Pemberton, et al., 2016; Pyecroft et al., 2007). DFT1 and DFT2 cell lines have been shown to share many characteristics of in vivo tumours (Caldwell et al., 2018; Patchett et al., 2019; Siddle et al., 2013), making these cell lines a useful in vitro study system. Cells were maintained in RPMI‐1640 media with GlutaMAX (Gibco, 61870036) supplemented with 10% heat‐inactivated FBS (Gibco, 10,500‐064) and 50 μg/mL penicillin/streptomycin (Gibco, 15,070,063) or 50 μg/mL of gentamicin (Sigma, G1397) at 35°C and 5% CO2. Upon reaching 80–90% confluency, cells were detached using TrypLE Express (Gibco, 12,605,010) and passaged 1:3. Cells were maintained below Passage 30. DFT1 cell lines were tested for mycoplasma as described in (Stammnitz et al., 2018). DFT2 cells were tested for mycoplasma using the MycoAlert mycoplasma testing kit (Lonza, LT07‐418) when they entered the laboratory and were passaged in a mycoplasma‐free tissue culture facility after testing.

### 2.2 Transduction (green fluorescent protein cell lines)

Distinguishing DFT1 and DFT2 cells using flow cytometry based solely on their size and shape is difficult; thus, we labelled one of these cell lines with green fluorescent protein (GFP), allowing us to distinguish co‐cultured tumour cells reliably. The DFT1‐GFP (4906‐GFP) and DFT2‐GFP (RV‐GFP) cell lines were established using lentiviral transduction. HEK293T cells were used to produce lentiviral particles with PLKO‐GFP (pLKO_TRC001), psPAX2 and pMD2.G (provided by N. Divecha). Lentivirus particles were transduced into 4906 and RV cells. Following transduction, approximately 10^5^ cells expressing high levels of GFP relative to untransduced control cells were sorted by fluorescence‐activated cell sorting (FACS) on a BD FACS Aria II using the gating strategy presented in Figure S1. These sorted cells were then cultured for a further 2 weeks before being sorted for a second time to establish a geneous cell line with stable and high expression of GFP and remove cells which were not expressing GFP.

### 2.3 Direct co‐cultures

DFT1 and DFT2 cells were cultured in 12‐well plates (Corning, 3513) for 14 days. Cells were plated in 1 mL of culture media per well (for a concentration of 10^5^ cells/mL of media), which was replaced every 3 days. Triplicate wells were harvested daily and counted on a Guava® easyCyte™ model 6HT. The following culture conditions with varying DFT1 to DFT2 ratios at the start of the experiment were performed: giving an advantage to DFT1 (70–80% DFT1), giving no advantage to either cell line (50–60% DFT1), giving an advantage to DFT2 (30–40% DFT1) and monoculture controls (100% DFT1 or 100% DFT2). A total number of 10^5^ cells per well were plated, meaning a 50:50 co‐culture will start with 0.5 × 10^5^ DFT1 cells and 0.5 × 10^5^ DFT2 cells, while a monoculture will start with 10^5^ cells of either DFT1 or DFT2. This experiment was performed in duplicate, alternating the use of one GFP cell line and one unlabelled cell line (i.e., DFT1‐GFP was co‐cultured with DFT2, and DFT1 was co‐cultured with DFT2‐GFP) to eliminate potential effects of the GFP‐transduction process and selection by FACS on cell growth. Imaging of the cells during one representative direct co‐culture experiment can be found in Figure S5.

### 2.4 Transwell co‐cultures

DFT1 and DFT2 cells were co‐cultured using 12‐well transwell plates (Corning, CLS3460). Transwells allow cells to remain in two compartments separated by a semi‐permeable membrane, permitting small molecules to be exchanged but keeping cell lines separated. Monocultures (cells of a same DFT grown in the inserts and wells) were compared to co‐cultures (cells of one DFT in the inserts and of the other DFT in the wells). Cells were plated at a density of 0.5 × 10^5^ cells/insert and 10^5^ cells/well (the inserts having a surface about two times smaller than the wells), for both monocultures and for co‐cultures. Cells were plated in 0.5 mL of culture media in the inserts and 1 mL of media in the wells (for a final concentration of 10^5^ cells/mL of media) which was replaced every 3 days. Duplicate wells were harvested and analysed on a Guava® easyCyte™ model 6HT every 2 days. Only cells from the wells were counted to avoid any effect of the surface size and type of the transwell inserts on cell growth. The experiment was performed in duplicate.

### 2.5 Flow cytometry

Cells were incubated in the dark on ice for 15 min with 1 μg/mL propidium iodide as a live/dead marker. Cells were run on a Guava® easyCyte™ model 6HT, and data were analysed using the CytoExploreR R package (Hammil, 2021). Gating performed first selected cells from debris (FSC‐Height vs. SSC‐Height), then singlets from doublets (SSC‐Area, SSC‐Height), live from dead cells (FSC vs. RED), and, for the direct co‐cultures, GFP‐positive cells from GFP‐negative cells (FSC vs. GRN; a representative gating strategy is shown in Figure S2). The number of cells in each well was calculated as follows: (number of gated events/volume analysed by the flow cytometer) × volume of cells per well (1 mL).

### 2.6 Growth rate and carrying capacity estimation

A logistic differential equation (Equation 1) was used to represent the growth of DFT1, DFT1‐GFP, DFT2 and DFT2‐GFP cell lines as this model has been shown to accurately describe DFT cell growth in vitro in a preliminary analysis (Gérard, 2020) and in vivo (Hamede et al., 2017). In Equation (1), *N* represents the number of cells, *r* represents the per capita cellular growth rate (per day), and *K* represents the maximum number of cells the space and resources can accommodate (i.e., carrying capacity).

(1) $$\frac{\mathrm{dN}}{\mathrm{dt}}=\mathrm{rN}\left(1-\frac{N}{K}\right)$$

A grid search method was used to simulate growth curves using 10,000 combinations of the growth rate *r* (ranging from 0.01 to 1, with a step of 0.01 per day) and *K* (ranging from 10^4^ to 10^6^, with a step of 10^4^ cells). *N* was initialized with the number of cells at day 1 of the experiment, that is, once cells have had time to attach to the surface of the plate and unattached dead cells were removed, to meet the model's assumption that cell population grows with time. The adequacy of each combination of parameter values was then assessed on each replicate growth curve of the DFT cells in monoculture (direct co‐cultures) and in co‐culture (transwell co‐cultures) by calculating the root mean square error (RMSE) between the simulated and the observed population dynamics of each setting. Parameters from simulations with the best fit (i.e., lowest RMSE) to the experimental data were then selected. Median values of *r* and *K* were compared between DFT1 and DFT2 cell lines using a Wilcoxon rank‐sum test with continuity correction. The *r* and *K* parameters were also estimated for DFT and DFT‐GFP cell lines, which showed that transduction appears to have lowered the carrying capacity of DFT1‐GFP cells (Figure S3).

### 2.7 Competition coefficient estimation

The two‐species competition Lotka–Volterra equations (Equations 2 and 3; Lotka, 1910; Volterra, 1927) were used to quantify competitive interactions between DFT1 and DFT2 cells. *N*_*i*_, *r*_*i*_ and *K*_*i*_ represent the number of cells, growth rate and carrying capacity for DFT1 (*i* = 1) and DFT2 (*i* = 2). The *α* parameter represents the competitive impact of DFT1 on the growth rate of DFT2, and vice versa for 𝛽. If a competition coefficient (*α* or 𝛽) is close to zero, a tumour line does not influence the growth of the other; if *α* or 𝛽 is bigger than 1, a tumour line negatively impacts the growth of the other; and if *α* or 𝛽 is lower than zero, a tumour line facilitates the growth of the other. Hence, *α* and 𝛽 inform the type of interaction between DFT1 and DFT2: competition (alpha and beta are positive), mutualism (alpha and beta are negative), commensalism (alpha is negative and beta is close to or equal to zero, or vice versa) or parasitism (alpha is positive and beta is negative, or vice versa).

(2) $$\frac{dN_{1}}{\mathrm{dt}}=r_{1}N_{1}\left(1-\frac{N_{1}+{\mathrm{\alpha N}}_{2}}{K_{1}}\right)$$

(3) $$\frac{dN_{2}}{\mathrm{dt}}=r_{2}N_{2}\left(1-\frac{N_{2}+{\mathrm{\beta N}}_{1}}{K_{2}}\right)$$

Again, a grid search method was used to simulate growth curves using 10,404 combinations of *α* and 𝛽 (each ranging from −100 to 100, with a step of 1). Mean values of *r*_1_, *r*_2_, *K*_1_ and *K*_2_ estimated on the monocultures, as described above, were fixed in the equations to only estimate the competition coefficients. The estimation of these parameter values was performed using the same approach than previously but using this time the population dynamics of both DFTs in direct co‐culture. Median values of *α* and 𝛽 were compared using a Wilcoxon rank‐sum test with continuity correction. Model fitting and statistical analyses were performed in R (R version 4.1.3; R Core Team, 2023).

### 2.8 Predicting competition outcome

Competition outcome of the Lotka–Volterra model can be predicted by examining zero‐growth isoclines of the two competing species, for example (Pascual & Kareiva, 1996). Briefly, the number of cells at which the DFT1 or DFT2 population stops growing can be found by solving for *dN*_*i*_/*dt* = 0. The trajectory of both populations can then be represented on a phase diagram in which the zero net growth isoclines are given by Equations 4 and 5. The coordinates of the isoclines correspond to the intercepts of both axes (i.e., [*N*_1,*t*_ = 0, *N*_2,*t*_ = 0]). DFT1's zero net growth isocline has the coordinates [0, *K*_1_/*α*] and [*K*_1_, 0], and DFT2's [0, *K*_2_] and [*K*_2_/𝛽, 0]. From these, we can determine the following outcomes: one of the tumour lines always outcompetes the other, competition outcome depends on initial conditions (i.e., the number of DFT1 and DFT2 cells at the start of the experiment), or both tumour lines coexist (Table 1).

(4) $$N_{1}=K_{1}-\alpha N_{2}$$

(5) $$N_{2}=K_{2}-\beta N_{1}$$

**TABLE 1:** Outcomes of the Lotka–Volterra two‐species competition model depending on the relationship between carrying capacities and competition coefficients.

DFT2 outcompetes DFT1	$K_{1}>\frac{K_{2}}{\beta }$	$K_{2}<\frac{K_{1}}{\alpha }$
DFT1 outcompetes DFT2	$K_{1}<\frac{K_{2}}{\beta }$	$K_{2}>\frac{K_{1}}{\alpha }$
One DFT outcompetes the other depending on initial conditions	$K_{1}>\frac{K_{2}}{\beta }$	$K_{2}>\frac{K_{1}}{\alpha }$
DFT1 and DFT2 coexist	$K_{1}<\frac{K_{2}}{\beta }$	$K_{2}<\frac{K_{1}}{\alpha }$

## 3 RESULTS

### 3.1 DFT2 shows a higher growth rate but lower carrying capacity than DFT1

**FIGURE 2:**
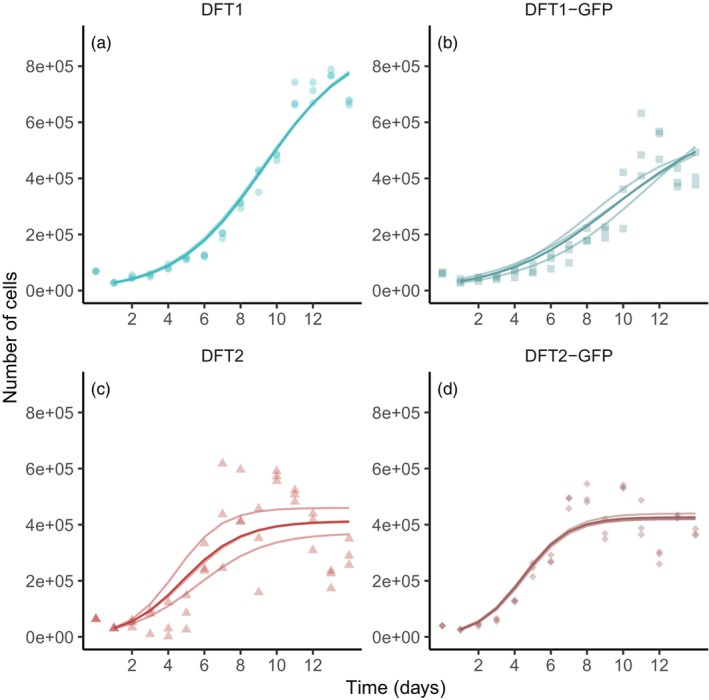
Direct co‐cultures – growth curves for DFT cells in monoculture. Points represent experimental cell counts for each day across three replicates. (a) DFT1 and (b) DFT1‐GFP are shown in blue, and (c) DFT2 and (d) DFT2‐GFP are shown in red. Light lines represent the best fit logistic model obtained using a grid search to estimate growth rates and carrying capacities shown in Figure 3a,b. Dark lines represent the averaged best fit model.

**FIGURE 3:**
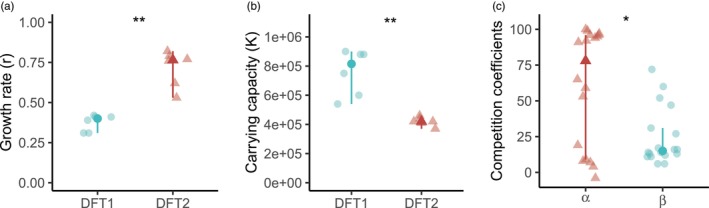
Direct co‐cultures – DFT2 shows a higher growth rate but lower carrying capacity than DFT1. Optimized (a) growth rates (per day) and (b) carrying capacities (number of cells) obtained by fitting a logistic model through DFT monocultures. (c) Optimized competition coefficients (*α* being the effect of DFT2 on the growth rate of DFT1 and 𝛽 the effect of DFT1 on the growth rate of DFT2) obtained by fitting a Lotka–Volterra competition model through DFT co‐cultures. DFT1 is shown in blue, and DFT2 is shown in red. All replicates are shown with median and 95% confidence intervals (calculated with the R MedianCI function from the DescTools package). Significance levels: ns *p* > 0.05, *p <= 0.05, **p <= 0.01, ***p <= 0.001. Dots represent DFT1 parameters, and triangles represent DFT2 parameters.

Visual examination of the DFT1 and DFT2 cells revealed morphological differences: DFT2 cells have a neuron‐like phenotype, similar to their Schwann cell progenitor (Owen et al., 2021), while DFT1 cells have a rounder shape (Figure 1b,c). To test the hypothesis that DFT2 is a better competitor than DFT1, we first grew DFT1 and DFT2 cell lines in monoculture (Figure 2). Fitting logistic growth curves to these experimental data showed that the two tumour cell lines favour different growth strategies in vitro. When reaching exponential growth, DFT2 cells grew nearly twice as fast as DFT1 cells (*p* < 0.005), with a median growth rate (*r*) of 0.76 per day compared to 0.40 per day for DFT1 (95% CI [0.53, 0.82] and [0.31, 0.42], respectively; Figure 3a). Our analysis also revealed that although slower growing, DFT1 cells were able to sustain a significantly higher maximum population size (*K*) than DFT2 cells (*p* < 0.005), with a median carrying capacity of 8.15 × 10^5^ cells compared to 4.2 × 10^5^ cells for DFT2 (95% CI [5.4 × 10^5^, 9 × 10^5^] and [3.7 × 10^5^, 4.6 × 10^5^]), respectively (Figure 3b).

### 3.2 DFT2 outcompetes DFT1 in direct co‐cultures

**FIGURE 4:**
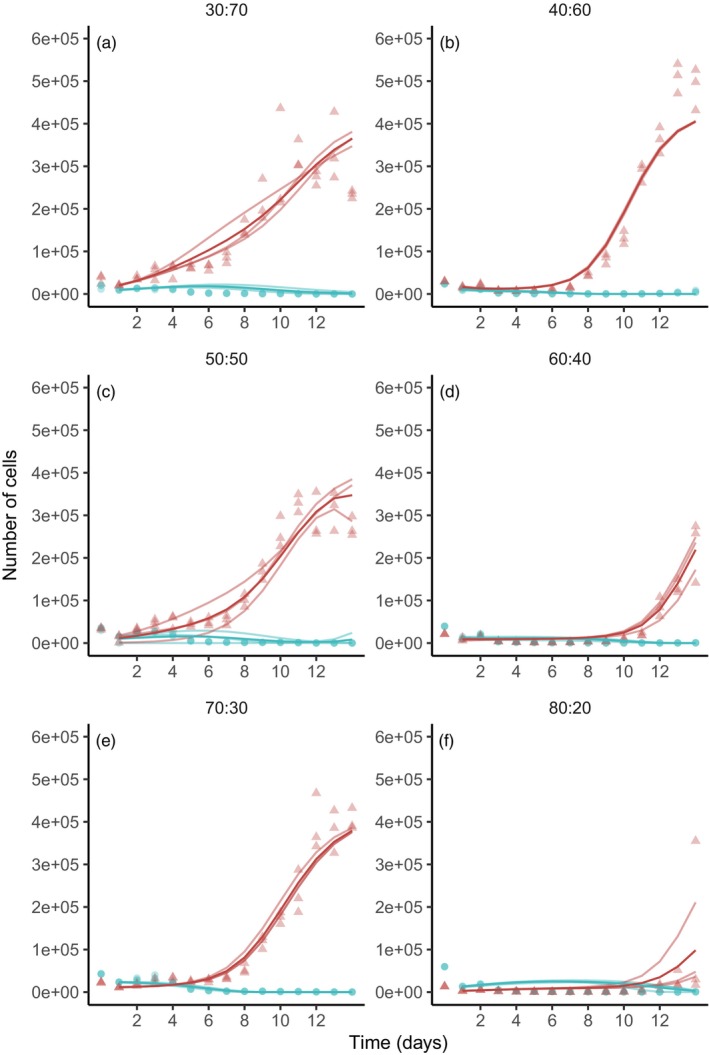
Direct co‐cultures – growth curves for DFT cells in co‐culture. Points represent experimental cell counts for each day across three replicates for two experiments with varying starting ratios of DFT1 and DFT2 cells (shown as DFT1:DFT2) and with two different combinations of GFP and non‐GFP cell lines: DFT1 and DFT2‐GFP shown in panels (b, d and f), and DFT1‐GFP and DFT2 in panels (a, c and e). DFT1 is shown in blue, and DFT2 is shown in red. Light lines represent the best fit two‐species Lotka–Volterra competition model obtained using a grid search to estimate the competition coefficients shown in Figure 3c. Dark lines represent the averaged best fit model.

We then established GFP‐labelled DFT1 and DFT2 cell lines in order to culture these transmissible cancer cells together and evaluate their competitive abilities. The co‐culture assays showed that, for any starting ratio of cells, DFT2 always reached much higher cell numbers than DFT1 after 14 days (Figure 4). When given a considerable disadvantage (starting ratio of 20% of DFT2 cells), after a slow growth likely due to low starting cell density, DFT2 outgrew DFT1 from day 12 onwards. In fact, in co‐culture, the DFT1 cell population decreases and never achieves exponential growth as it did in monoculture. These results were consistent independently of the combination of GFP and non‐GFP cell line used. Fitting a Lotka–Volterra competition model on the co‐cultures allowed us to quantify competition between the cell lines. Both competition coefficients were higher than 0 (*α* = 78 and 𝛽 = 15), indicating strong competition between both tumour lines (95% CI [9, 96] and [12, 31], respectively; Figure 3c). DFT2 cells negatively impacted the growth of DFT1 cells more than DFT1 cells impacted DFT2 (*α* > 𝛽; *p* < 0.05).

We subsequently used the carrying capacities, estimated from monocultures, along with these competition coefficients to predict whether DFT2 can also outcompete DFT1 in scenarios that were not tested in vitro (Table 1; Equations 4 and 5). We found that DFT2 might not always outcompete DFT1 and that competition outcome depends on the initial number of DFT1 and DFT2 cells. Indeed, using simulations we were able to show that DFT1 was able to outcompete DFT2 when the starting ratio of tumour cells is 90% DFT1 and 10% DFT2 (Figure S4).

### 3.3 Intertumoral competition is only observed in direct co‐cultures

**FIGURE 5:**
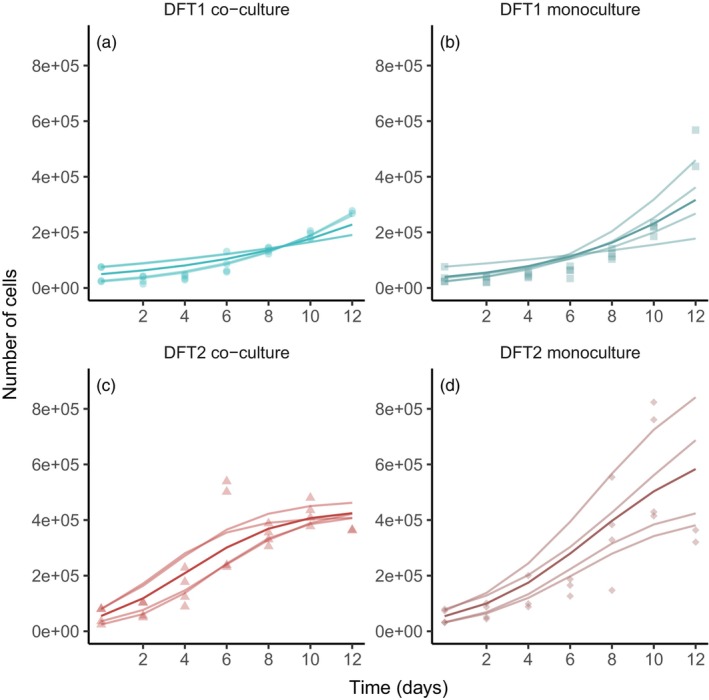
Transwell co‐cultures – growth curves for DFT cells in transwell cultures. Points represent experimental cell counts over time across two replicates and two experiments. DFT1 is shown in blue, and DFT2 is shown in red. Light lines represent the best fit logistic model obtained using a grid search to estimate growth rates shown in Figure 6. Dark lines represent the averaged best fit model.

**FIGURE 6:**
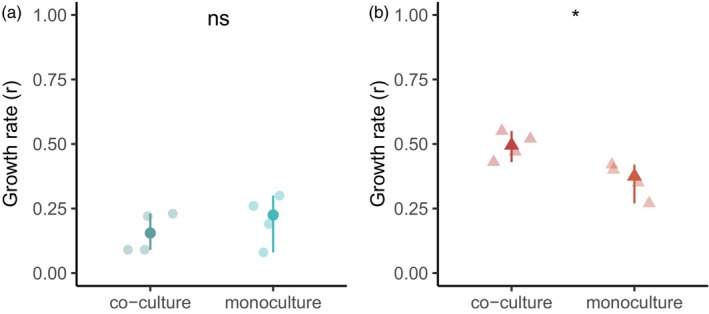
Transwell co‐cultures – intertumoral competition is only observed in direct co‐cultures. Optimized (a) growth rates (per day) for DFT1 cells and (b) DFT2 cells in transwell monocultures and co‐cultures obtained by fitting a logistic model. DFT1 is shown in blue, and DFT2 is shown in red. All replicates are shown with median and 85% confidence intervals (calculated with the R MedianCI function from the DescTools package). Significance levels: ns *p* > 0.05, *p <= 0.05, **p <= 0.01, ***p <= 0.001. Dots represent DFT1 parameters, and triangles represent DFT2 parameters.

Finally, to obtain insight into potential mechanisms of competition, DFT1 and DFT2 cells were co‐cultured in transwells where cell lines were physically separated by a semi‐permeable membrane (Figure 5). If the previously observed competition outcome relies on mechanical stress (Gatenby & Brown, 2020) caused by DFT2's faster growth rate, we expect DFT1 cells in co‐culture with DFT2 to grow as well as they would in monoculture. Indeed, there was no significant difference in growth rate between monocultured and co‐cultured DFT1 cells (*p* = 0.663) indicating that DFT2 did not negatively impact the growth of DFT1 cells in a transwell setting where it was unable to cause mechanical stress (Figure 6a). Interestingly, there was a slight increase in the growth rate of DFT2 cells in transwell co‐culture relative to monoculture (*p* = 0.030; Figure 6b).

## 4 DISCUSSION

This study provides the first empirical in vitro assessments of growth dynamics and competition between two transmissible cancer cell lines that have originated from a single host species. Transmissible cancers are rare (see Dujon et al., 2021), but their epidemiological and evolutionary dynamics are relevant to understand how species respond to novel disease threats. As competitive interactions between DFT1 and DFT2 may influence both malignant evolutionary trajectories and the host population, it becomes urgent to explore the modalities and the outcomes of the competition between these two transmissible cancers. We therefore conducted in vitro and modelling experiments to establish the competitive capabilities of a representative cell line of DFT1 (4906 (Pyecroft et al., 2007)) and DFT2 (RV (Pye, Pemberton, et al., 2016)).

For a new cancer to successfully emerge in an already occupied ecological niche (i.e., cancers on the same organ and/or same host in the context of transmissible cancers), that new cancer would need superior competitive abilities (Tissot et al., 2022; Ujvari, Gatenby, & Thomas, 2016). DFT2 appeared in a devil population where DFT1 was already present (Pye, Pemberton, et al., 2016). Hence, only fast‐growing DFT2 clones and/or clones that could suppress DFT1, survived. Such faster growing DFT2 tumours would have shorter latency periods and/or increased transmission potential, ultimately outcompeting slower DFT1 tumours. Our in vitro results support this theoretical scenario. Firstly, measurements of cellular growth rate demonstrate that DFT2 grows twice as fast as DFT1. Secondly, direct in vitro competition assays show that DFT2 cells always outcompete DFT1 cells, and transwell competition assays that DFT2 cells grow faster in co‐culture with DFT1 cells than in monoculture. Lastly, DFT2's faster growth rate implies an increased cell division rate, higher potential for DNA replication errors, and hence accumulation of mutations; a pattern observed by (Stammnitz et al., 2023), who found that DFT2 tumours have higher mutation rates compared to DFT1 tumours. Higher genetic variation of DFT2 tumour lines may also provide them with greater opportunity to evolve (Fisher, 1930) and adapt in the competition with DFT1. Taken together, our results align with in vivo observations in the field where the range and prevalence of DFT2 is increasing in the d'Entrecasteaux Peninsula (James et al., 2019) despite co‐occurring with DFT1. The low devil population density in the Peninsula (James et al., 2019) could not have provided enough traction (i.e., contact rates and transmission probabilities) for one disease to outcompete the other yet. Our in vitro results point towards DFT2 being a better competitor on a within‐host scale; however, care should be taken before generalizing them to between‐host dynamics in the wild. Indeed, we did observe more variation between replicates for DFT2, perhaps reflecting the shorter amount of time that these cells have been in culture compared to DFT1. Thus, here we present the results of two representative DFT1 and DFT2 cell lines (grown in cell culture) which might not adequately represent the current most prevalent DFT cancers in the wild.

Interestingly, we did not observe competitive exclusion of DFT1 in the transwell co‐cultures, in contrast to the direct co‐cultures. The transwell assays also showed that DFT2 cells grow faster when co‐cultured with DFT1 cells compared to monocultures, suggesting that, in this setting, DFT1 could promote the growth of DFT2. Hence, we cannot exclude that more complex interactions are at play. For instance, DFT cells could be switching phenotype in response to environmental pressures. Non‐small cell lung cancer cells have been shown to switch between altruistic and competitive strategies in response to stressors in their microenvironment, such as other faster growing cell types or chemotherapy (Nam et al., 2021). In our transwell experiments, neither cell lines had to compete for space; thus, DFT2 could have benefited from the presence of DFT1, for example, through the release of sharable resources (such as growth factors; Axelrod et al., 2006). Conversely, when space is shared and resources limited, the faster growing DFT2 cells may be exerting a force (i.e., mechanical stress) on DFT1. This results in mechanical cell competition, a phenomenon whereby mechanical stress triggers cell elimination through excessive stretching or compression (Brás‐Pereira & Moreno, 2018; Matamoro‐Vidal & Levayer, 2019), which may explain DFT2's success in direct co‐culture (Figure S5). Future studies should focus on defining these competitive interactions in vivo, through the collection and analysis of field data from devils co‐infected with both DFT1 and DFT2 tumours. In vivo tumour growth rates could then be estimated (as in (Gause & Witt, 1935)), along with competition coefficients (as in the present study), and compared between tumours in close proximity (i.e., competing for space) and tumours located further away from each other.

An interesting theory could be that DFT2 could have evolved the ability to adapt its growth strategy depending on the presence or absence of DFT1, a capacity that DFT1 is lacking as it achieved most of its evolution without the presence of other transmissible cancers. Although our experiments and modelling did not investigate cellular hysteresis, that is, long‐lasting transgenerational changes in cellular physiology (Roemhild et al., 2018) which could have resulted from DFT1 and DFT2 sharing the same environment, further experiments undertaking subsequent rounds of co‐culture using daughter cells from primary experiments could help explore this hypothesis.

The cellular origins of DFT1 and DFT2 could explain their different competitive capacities. DFT1 originated from a well‐differentiated myelinating Schwann cell (Owen et al., 2021) a cell type that usually exits the cell cycle and ceases growth (Tikoo et al., 2000; Yamauchi et al., 2004); however, DFT2 originated from a less differentiated immature or repair Schwann cell (Owen et al., 2021; Patchett et al., 2019), a cell type that retains the ability to proliferate (Tikoo et al., 2000; Yamauchi et al., 2004). Previous work (Patchett et al., 2019) found that, in comparison with DFT1, DFT2 transcriptomes were enriched in genes linked to cell migration consistent with a repair Schwann cell origin. Given the increased migratory capacity of repair Schwann cells relative to differentiated myelinating Schwann cells (Chen et al., 2019), it would be interesting to investigate whether this phenotype has been retained by DFT2. Human Schwann cell tumours emerging from less well‐differentiated progenitor cells have also been more strongly associated with aggressive, malignant and metastatic disease (Carroll, 2012; Chen et al., 2014; Le et al., 2011), which could explain DFT2's growth rate advantage. In addition, DFT2 cells' neuron‐like morphology (Figures 1b,c and S5) also suggests that they may occupy more space in in vitro culture and such could explain our findings of DFT1 cells being able to reach significantly higher maximum cell densities compared to DFT2.

Although our experiments were conducted in vitro, in the absence of hosts, below we provide some hypotheses to how these cancers' growth dynamics could not only influence how they compete for resources but also impact between‐host dynamics. Based on the observation in cell cultures, we propose that DFT1 may present a slower growth rate and higher carrying capacity that could be consistent with optimized transmission following decades of evolution with its host (i.e., the virulence trade‐off hypothesis (Lipsitch & Moxon, 1997)). As mentioned above, DFT2 could benefit from its faster growth rate, which could result in a shorter latency and increased transmission, in its competition with DFT1. In the long term however, this could result in faster host mortality and reduced transmission, explaining the currently observed slow expansion of DFT2 relative to DFT1 in the wild (James et al., 2019). Only three cases of DFT1 and DFT2 co‐infections have been reported so far (R. Hamede, personal communication; James et al., 2019; Kwon et al., 2018), despite the tumours co‐occurring at local and regional scales. This pattern of infection could be due to the long latency of the disease (up to 12 months), thus co‐infected devils might succumb to a first DFT infection before becoming symptomatic with a second DFT (James et al., 2019). Finally, DFT2 may avoid competition in hosts with well‐developed DFT1 tumours that could outcompete the incoming DFT2 cells (as shown by our modelling), by seeding in other bodily locations (the body as opposed to the head), as proposed by (James et al., 2019). While these data provide an interesting foundation for assessing competitive interactions between these two tumours, in vivo observations are necessary to validate or reject any hypotheses related to how these interactions occur in the wild.

Like the evolution of heterogeneous tumours in single organisms, the evolution of competitive interactions between DFT1 and DFT2 is complex and has unlikely reached evolutionary stability. Many factors, including tumour cell lineages, their geographic overlap, the anatomical position of the tumours and their virulence and health impact on their host, will determine long‐term evolutionary outcomes in these competing cancer epidemics. Given the detrimental effect of DFT1 on the devil population, the emergence of a new, potentially more competitive tumour raises concerns for the future of the host species and highlights the importance of studies defining competitive interactions between transmissible cancers in wild populations. As the level of virulence can differ across pathogens, and depend on the interaction between host and pathogens, applying evolutionary theories to in vitro experiments and mathematical modelling can provide a powerful framework to understand the extent to which DFT1 and DFT2 will harm their hosts and how their virulence may change over time.

Integrating evolutionary concepts into intervention strategies can lead to dramatic progress in mitigating the impact of diseases (Olesen, 2022). Here, we generated essential information on the growth rate and competitive potential of DFT1 and DFT2 tumours in vitro, data that is logistically difficult to obtain in vivo, and these results have been incorporated into modelling approaches to predict disease dynamics and epidemiology. Our results provide a preliminary framework for investigating the proliferation dynamics and underlying mechanisms of transmissible tumours, information that leads to better understanding of disease transmission, progression and outcomes. Previous studies have developed models to predict the epidemiological outcomes of DFT1 across wild devil populations (Cunningham et al., 2021). However, these models lacked in vivo and in vitro data on tumour kinetics that would be essential for complete understanding of the ecology and the epidemiology of transmissible cancers, as well as for the conservation of Tasmanian devils. Here, we have generated in vitro kinetic data on these tumours, which has been effectively incorporated into epidemiological models to predict competitive outcomes between the two DFTs. While the in vitro data generated here provide interesting insight into the competitive interactions between these tumours, the ongoing monitoring of the Tasmanian devil populations both in the d'Entercasteaux Peninsula and across Tasmania, as well as the generation of in vivo kinetic data, is essential to continue to understand the competition between DFT1 and DFT2, to evaluate epidemiological dynamics and to elaborate conservation strategies for the species. The results of our study will be used to improve the management of these extinction threatening diseases and, on a broader scale, to provide new insights and avenues for the conservation of species affected by wildlife diseases.

## CONFLICT OF INTEREST STATEMENT

The authors declare no conflicts of interest.

## Supporting information


Figure S1.

Figure S2.

Figure S3.

Figure S4.

Figure S5.

Figure Legents.

## Data Availability

Data for this study are available at the Dryad Digital Repository: https://doi.org/10.5061/dryad.3j9kd51s6.
