# Supplementary figures and images for: In vitro competition between two transmissible cancers and potential implications for their host, the Tasmanian devil
Source: Evol Appl. 2024 Mar 10;17(3):e13670. doi: 10.1111/eva.13670 (PMC10925828; doi:10.1111/eva.13670)

# BD FACSDiva 8.0.2

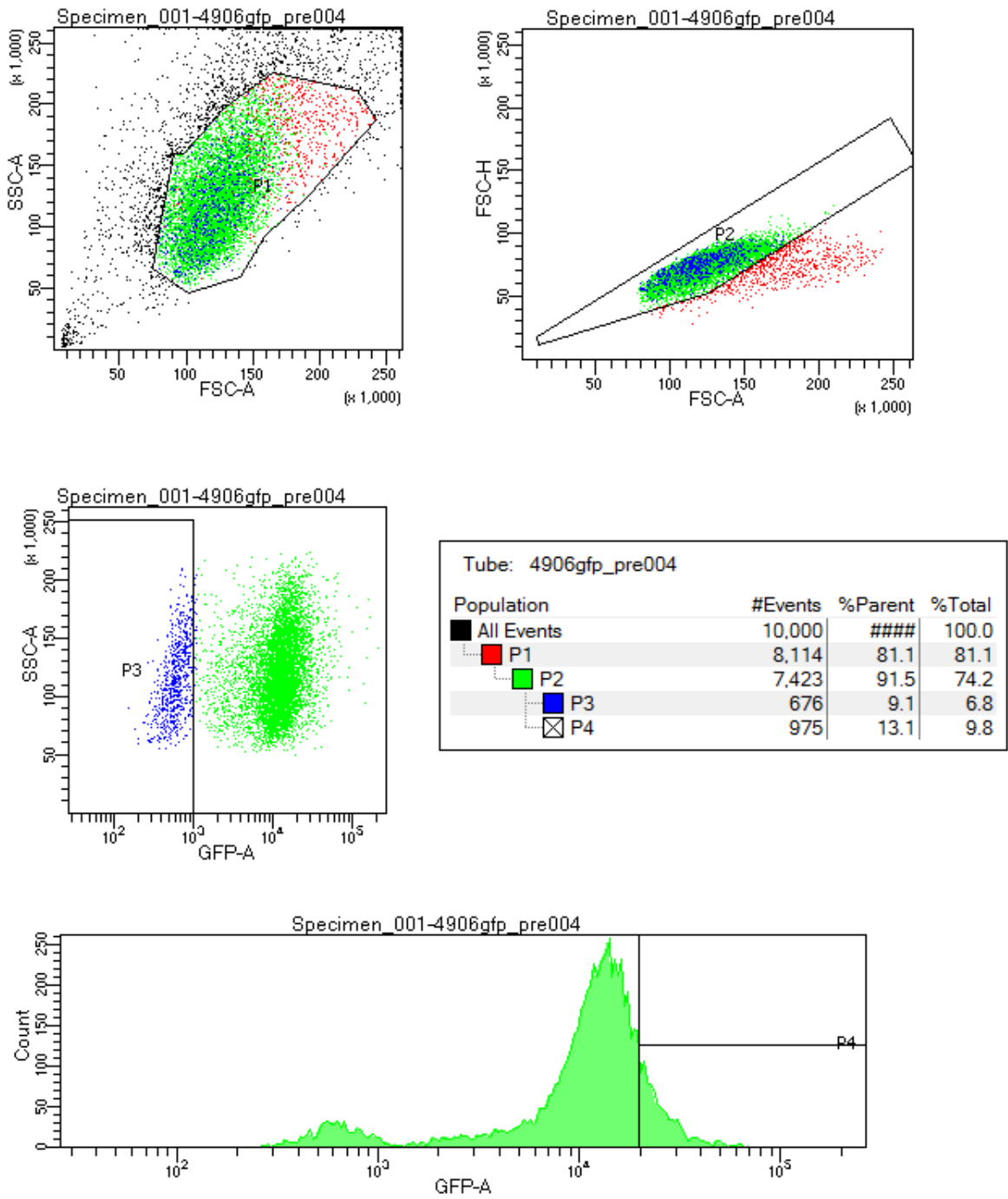

Supplement: Supplementary file 1 — Figure S1. [file EVA-17-e13670-s001.pdf]

# 5050\_d1\_1.fcs

## All Events

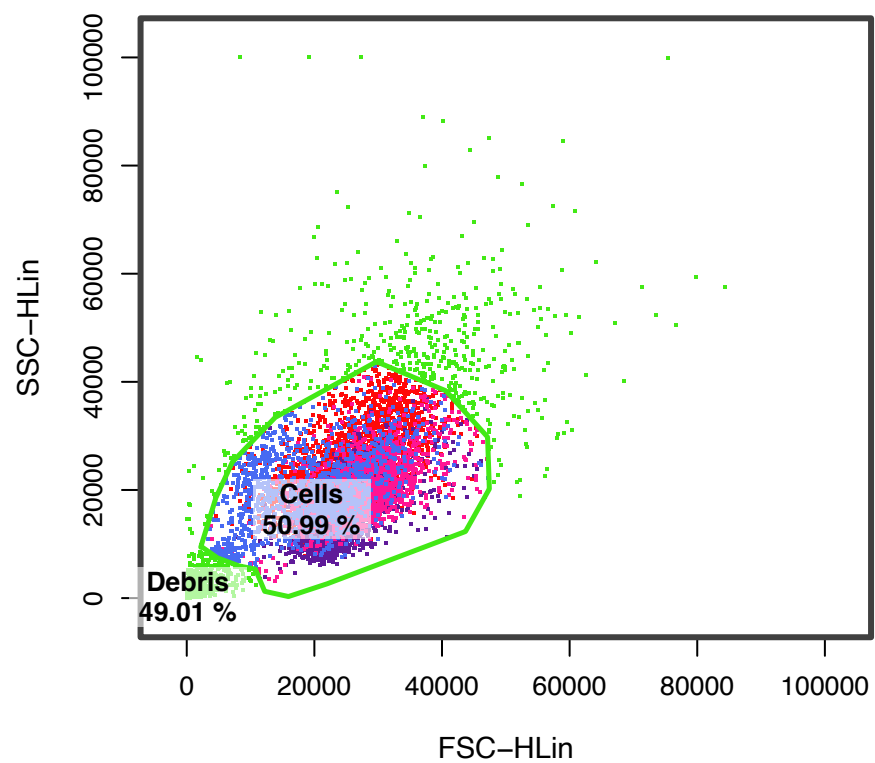

## Cells

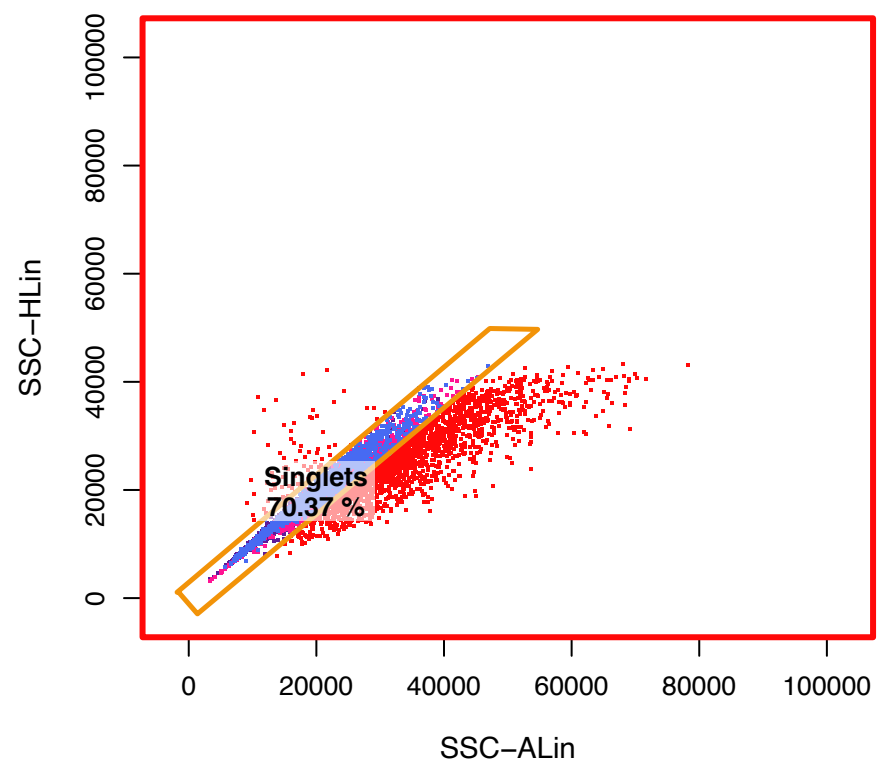

## Singlets

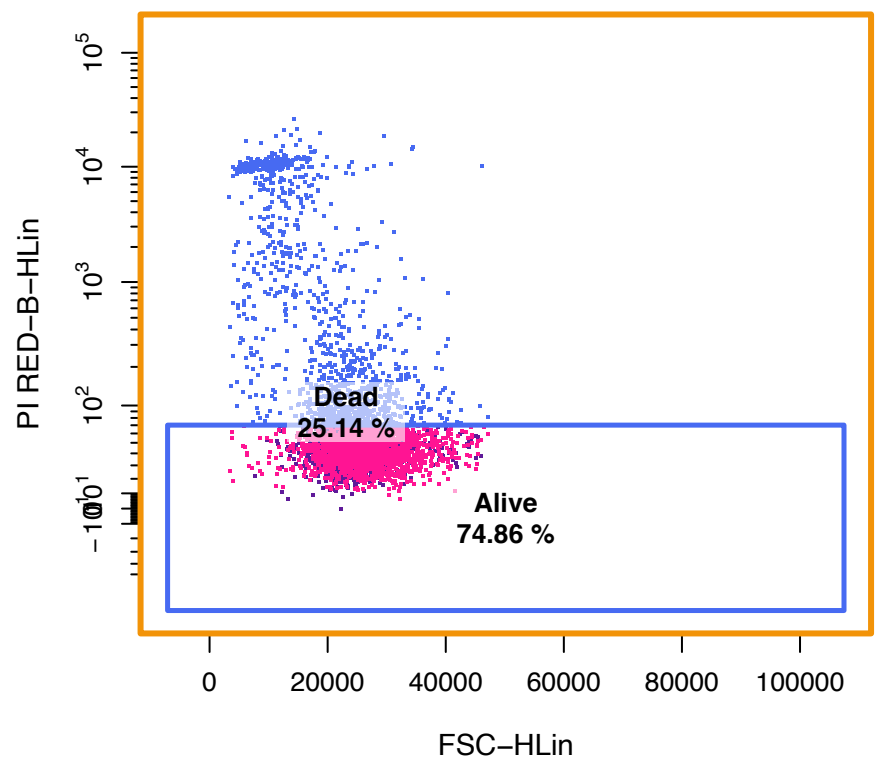

## Alive

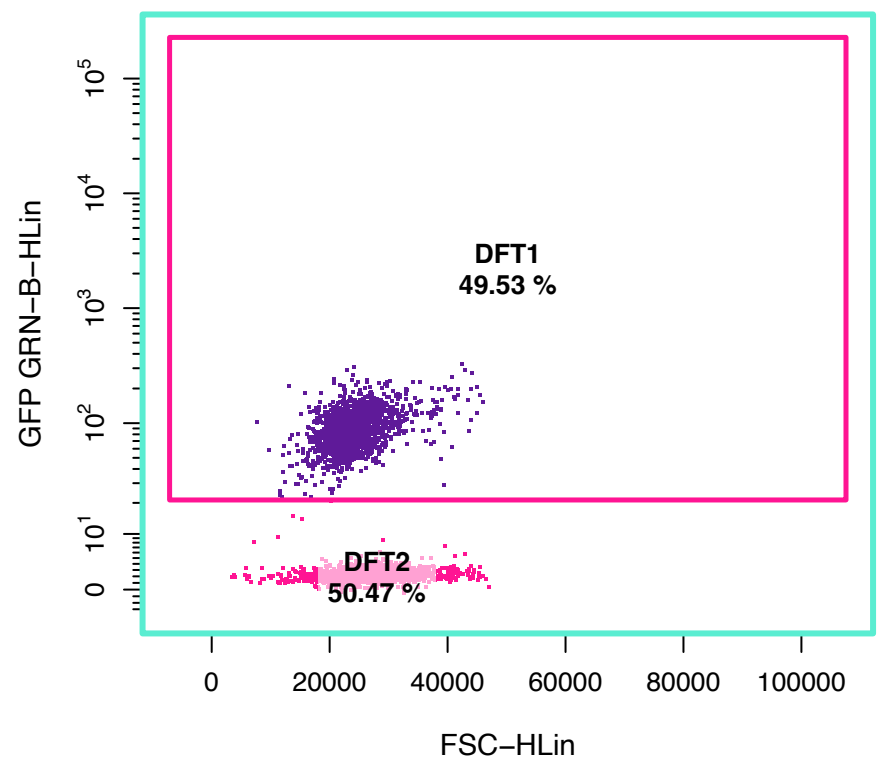

Supplement: Supplementary file 2 — Figure S2. [file EVA-17-e13670-s002.pdf]

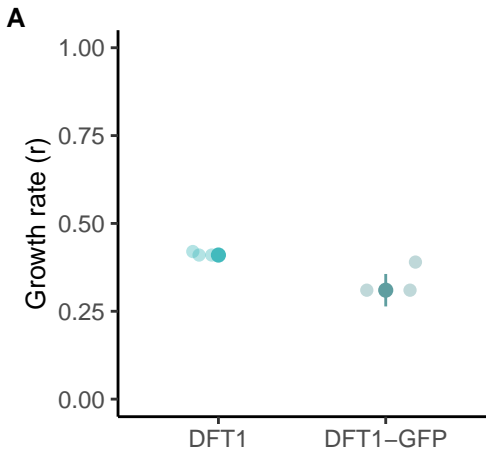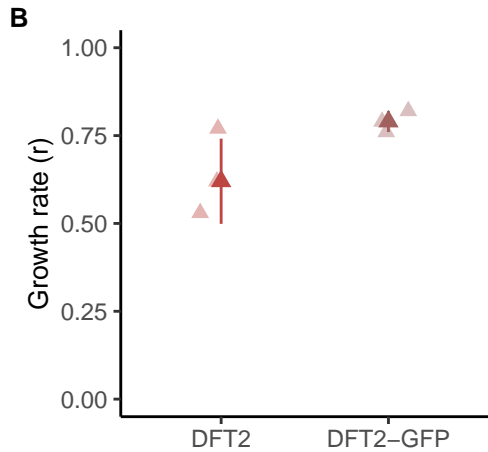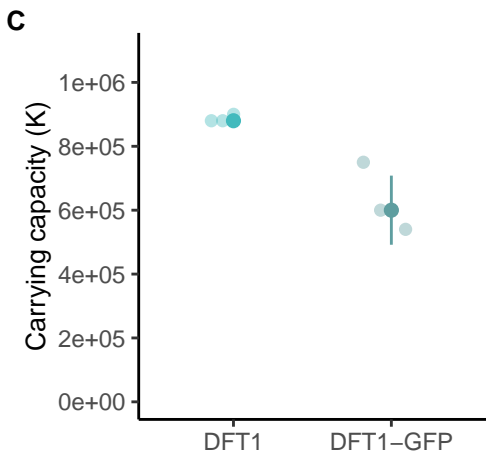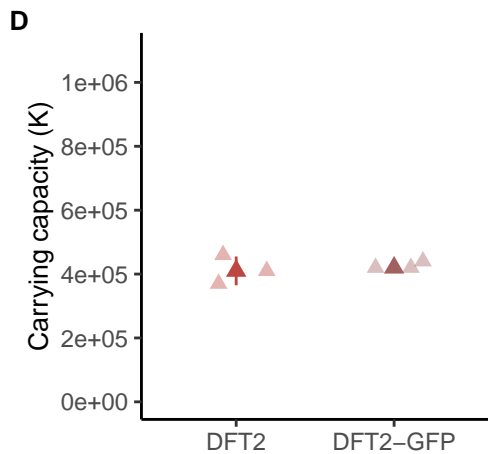

Supplement: Supplementary file 3 — Figure S3. [file EVA-17-e13670-s005.pdf]

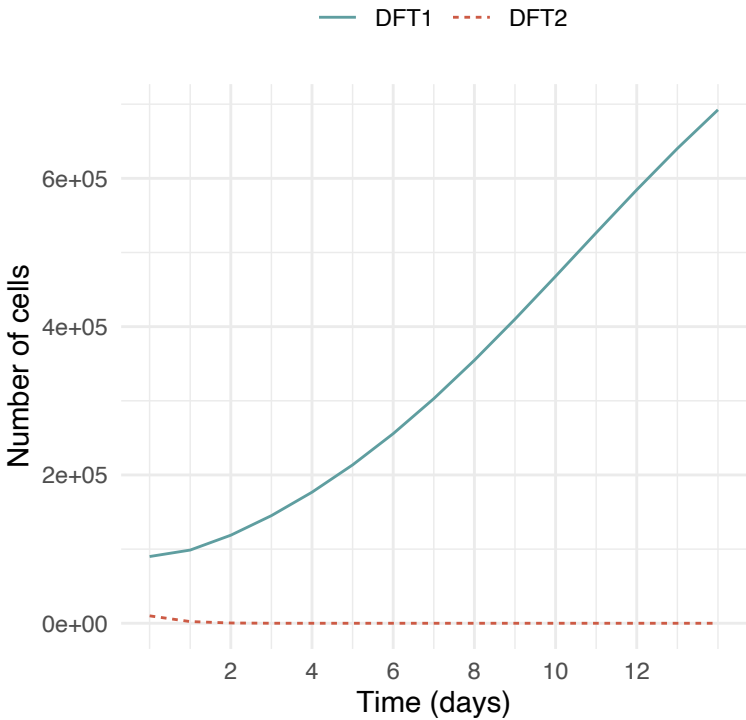

Supplement: Supplementary file 4 — Figure S4. [file EVA-17-e13670-s004.pdf]

Brightfield

Green fluorescence

Merged

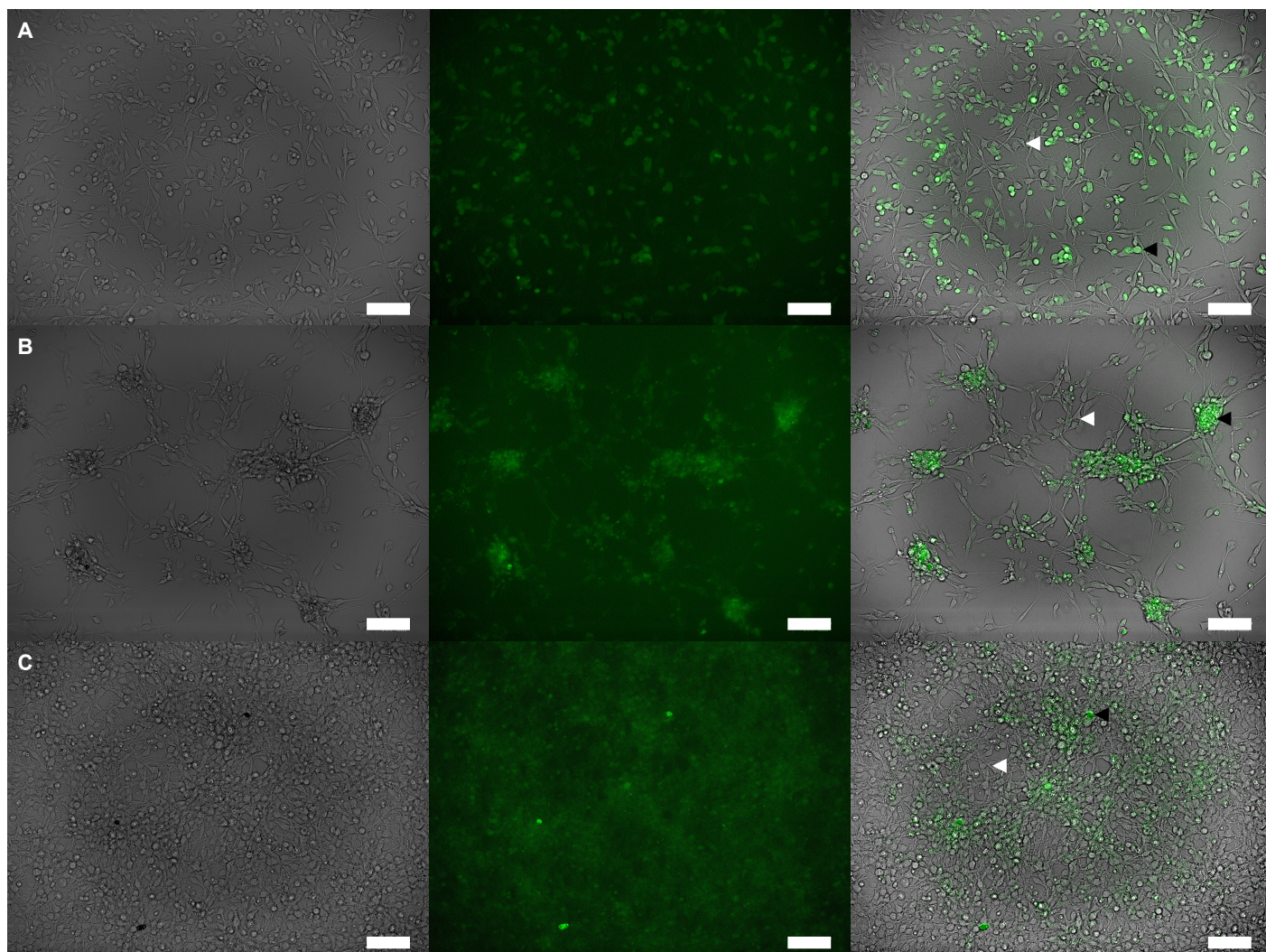

Supplement: Supplementary file 5 — Figure S5. [file EVA-17-e13670-s006.pdf]
