# Supplementary material for: In vitro competition between two transmissible cancers and potential implications for their host, the Tasmanian devil
Source: Evol Appl. 2024 Mar 10;17(3):e13670. doi: 10.1111/eva.13670 (PMC10925828; doi:10.1111/eva.13670)
Supplement: Supplementary file 6 — Figure Legents. [file EVA-17-e13670-s003.pdf]

Figure S1: Flow cytometry gating strategy used for the sorting of 4906-GFP cells. Gating performed first selected cells from debris (FSC-Area vs SSC-Area), then singlets from doublets (FSC-Area, FSC-Height) and high GFP expression from low GFP expression (GFP-Area vs SSC-Area, and then GFP-Area vs count).

Figure S2: Flow cytometry gating strategies. Gating performed first selected cells from debris (FSC-Height vs SSC-Height), then singlets from doublets (SSC-Area, SSC-Height), live from dead cells (FSC vs RED), and, for the direct co-cultures, GFP positive from GFP negative cells (FSC vs GRN).

Figure S3: Effect of transduction on cell growth. Optimised A-B) growth rates (per day) and C-D) carrying capacities (number of cells) obtained by fitting a logistic model through DFT monocultures. All replicates are shown with median and standard deviations. DFT1 is shown in blue, DFT2 is shown in red. Dots represent DFT1 parameters and triangles DFT2 parameters.

Figure S4: Example of simulation where DFT1 outcompetes DFT2. DFT1 is shown in blue, DFT2 is shown in red. Median values of all estimated parameters were used, i.e. r1 = 0.4, r2 = 0.76, K1 = 8.15x105, K2 = 4.2x105, α = 78 and β = 16. Initial number of cells at the start of the simulation: N1 = 9 x105 and N2 = 105.

Figure S5: Representative microscopy images of a direct co-culture between DFT1-GFP and unlabelled DFT2 cells. Starting ratio of 70% DFT1 cells and 30% DFT2 cells images at days 2 (A), 7 (B) and 13 (C). Scale bar: 100 µm. White arrow heads: DFT2; black arrow heads: DFT1-GFP.
